# Characteristics of Immunobiology in the Tumor Microenvironment- Development of Immunotherapies

**DOI:** 10.1155/2019/1513964

**Published:** 2019-03-24

**Authors:** Jian Song, Zenghui Teng, Weidong Cao

**Affiliations:** ^1^Institute of Physiological Chemistry and Pathobiochemistry, Cells-in-Motion Cluster of Excellence, University of Muenster, 48149 Muenster, Germany; ^2^Southwest Medical University, Luzhou, China; ^3^Navy General Hospital, Beijing, China

Immunotherapies might be, in the next few years, the most promising methods for cancer treatment. In the last decade, significant progress has been made in cancer immunotherapy, such as checkpoint blockers [1], adoptive cell transfer [2], and vaccine development [3]. However, there are still pain points, such as that only some cancer patients can benefit from their immunotherapy, and in clinical practice, cancer immunotherapies are associated with a range of immune-related adverse events. Therefore, a comprehensive understanding of cancer immunotherapy is highly desirable, to determine if a cancer patient is going to undergo immunotherapy and which immunotherapy strategy will be chosen.

Here, we are particularly proud to launch this special issue, which combines the research contributions of different research groups. Their research promotes our understanding of the role and function of immunotherapy in different tumors.

As a representative of emerging immunotherapies, PD-1/PD-L1 inhibitors have shown their effect in multiple cancer types [1]. In this issue, an article by M. Witkowska and P. Smolewski discussed the immunobiology of checkpoint blockade in lymphomas. Several PD-1 inhibitors demonstrated high efficacy in both Hodgkin and non-Hodgkin lymphoma patients. The side effects of checkpoint inhibitors have been as well statistically studied. They proposed that a combination of checkpoint inhibitors with other novel therapies might bring higher response rates in the lymphoma. In the following review article, J. Koury et al. had already given an overview and comparison of the three novel immunotherapies, including adoptive T cell transfer, checkpoint inhibitors, and bivalent antibodies. They outlined the method and the function as well as the major immunotherapeutic drugs developed in these 3 immunotherapies, which is pretty informative for treating a variety of cancers. Correlatively, the meta-analysis by Y. Zheng et al. showed that when combined with bortezomib or lenalidomide plus dexamethasone, monoclonal antibodies are superior to the histone deacetylase inhibitors in the treatment of relapsed/refractory multiple myeloma.

The original articles in this issue have been focusing on the diversity of PD-1 expression in the prognosis of different tumors. H. Tai et al. reported that tumor PD-L1 expression predicted diverse prognosis in Krukenberg tumors with different corresponding origins. In Krukenberg tumors from gastric carcinomas, positive tumor PD-L1 expression was associated with poor prognosis and in Krukenberg tumor from colorectal carcinomas, however, was associated with an improved prognosis. In contrast, in the primary gastric carcinomas, Y. Wang et al. found that positive PD-L1 tumor expression was associated with increased overall survival time and therefore an improved prognosis. The shifted PD-L1 correlation in the primary and metastasis tumors still requires further investigation, which is substantiated by the clinical study of Y.-J. Zhao et al. that PD-1 expression on CD4^+^ tumor-infiltrating lymphocytes indicated dysfunctional immune microenvironment. In the solid cancer, the PD-1/PD-L1 studies had also been investigated in the malignant peritoneal mesothelioma, where M. Tazzari et al. showed that PD-1/PD-L1 were employed by epithelioid malignant peritoneal mesothelioma in immunosuppression.

The final set of this issue features novel methods and concepts. W. Lv et al. generated an enhanced nanobubbles-Affibody for HER2^+^ breast cancer imaging using near-infrared fluorescent dye-IR783. The newly developed IR783-nanobubbles-Affibody is characterized with favourable HER2-targeting ability and bimodal imaging capability for breast cancer, which holds a great potential in molecular diagnosis. Using bioinformatics tools and databases, Z. Jin et al. disclosed the crucial genes and signaling pathways involved in bladder cancer and obtained fifteen module-related differentially expressed genes and their associated signaling pathways. The identified differentially expressed genes comprise several metastasis relevant genes and chemokines, which might be a valuable resource for the further studies of bladder cancer metastasis. Last but not the least, S. M. Toor and E. Elkord reported the influence of tumor microenvironment on the levels of granulocytic and immature myeloid cells. Comparing the patients with primary breast cancer and colorectal cancer, they concluded that although granulocytic myeloid cells were expanded in both breast cancer and colorectal cancer patients, the levels of these cells were significantly higher in the tumor microenvironment of colorectal cancer patients and that increased levels of circulating granulocytic myeloid cells are associated with poorly differentiated tumors in colorectal cancer patients.

In summary, considerable efforts have been made to understand the effects of immunotherapy on different tumor settings. We hope that the research in this special issue will inspire the understanding of immunotherapy, which can provide insights into the therapeutic strategies, help establish the therapy indications in various clinic treatment, and be valuable for the clinical studies.
